# Short-term effects of tropical cyclones on the incidence of dengue: a time-series study in Guangzhou, China

**DOI:** 10.1186/s13071-022-05486-2

**Published:** 2022-10-06

**Authors:** Chuanxi Li, Zhe Zhao, Yu Yan, Qiyong Liu, Qi Zhao, Wei Ma

**Affiliations:** 1 Department of Epidemiology, School of Public Health, Cheeloo College of Medicine, Shandong University, Jinan, China; 2 Shandong University Climate Change and Health Center, Jinan, China; 3 State Key Laboratory of Infectious Disease Prevention and Control, National Institute for Communicable Disease Control and Prevention, Chinese Center for Disease Control and Prevention, Beijing, China; 4 Department of Epidemiology, IUF-Leibniz Research Institute for Environmental Medicine, Düsseldorf, Germany

**Keywords:** Extreme weather event, Tropical storm, Typhoon, Dengue, Time series, Stratified analysis

## Abstract

**Background:**

Limited evidence is available about the association between tropical cyclones and dengue incidence. This study aimed to examine the effects of tropical cyclones on the incidence of dengue and to explore the vulnerable populations in Guangzhou, China.

**Methods:**

Weekly dengue case data, tropical cyclone and meteorological data during the tropical cyclones season (June to October) from 2015 to 2019 were collected for the study. A quasi-Poisson generalized linear model combined with a distributed lag non-linear model was conducted to quantify the association between tropical cyclones and dengue, controlling for meteorological factors, seasonality, and long-term trend. Proportion of dengue cases attributable to tropical cyclone exposure was calculated. The effect difference by sex and age groups was calculated to identify vulnerable populations. The tropical cyclones were classified into two levels to compare the effects of different grades of tropical cyclones on the dengue incidence.

**Results:**

Tropical cyclones were associated with an increased number of dengue cases with the maximum risk ratio of 1.41 (95% confidence interval 1.17–1.69) in lag 0 week and cumulative risk ratio of 2.13 (95% confidence interval 1.28–3.56) in lag 0–4 weeks. The attributable fraction was 6.31% (95% empirical confidence interval 1.96–10.16%). Men and the elderly were more vulnerable to the effects of tropical cyclones than the others. The effects of typhoons were stronger than those of tropical storms among various subpopulations.

**Conclusions:**

Our findings indicate that tropical cyclones may increase the incidence of dengue within a 4-week lag in Guangzhou, China, and the effects were more pronounced in men and the elderly. Precautionary measures should be taken with a focus on the identified vulnerable populations to control the transmission of dengue associated with tropical cyclones.

**Graphical Abstract:**

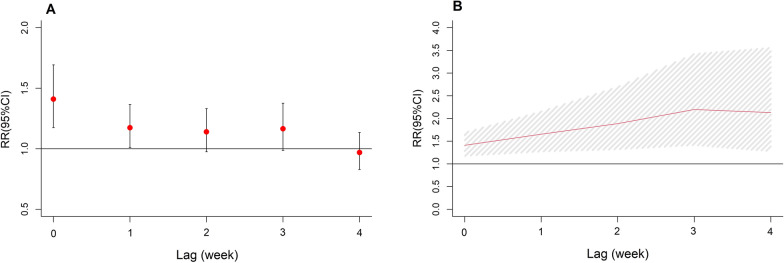

**Supplementary Information:**

The online version contains supplementary material available at 10.1186/s13071-022-05486-2.

## Background

In the context of global warming, extreme weather events (e.g. heat waves, floods, and tropical cyclones) are becoming increasingly frequent. Tropical cyclones are low-pressure eddies that occur on the tropical and subtropical oceans, threatening human safety and health in coastal areas worldwide with heavy rainfall, winds, and storm tides [1, 2]. The proportion of intense tropical cyclones and peak wind speeds of the most intense tropical cyclones are expected to increase at the global scale [3]. China has an extended coastline in the Western Pacific, which is frequently hit by tropical cyclones. Since 1980, an average of more than 11 tropical cyclones per year landed in China, and Guangdong is the province with the most frequent tropical cyclone landings [4].

Dengue is an acute mosquito-borne infectious disease mainly prevalent in tropical and subtropical areas [5]. The risk of dengue has being continuously rising over the past few decades because of accelerated global warming and urbanization as well as increased population density [6]. China suffers from a high disease burden of dengue. In 2014, there was an unprecedented dengue outbreak in China, with > 45,000 cases reported [7]. Since then, the incidence rate of dengue in China has been at a high level and dengue outbreaks have occurred every year in southern China. *Aedes aegypti* and *Aedes albopictus* are the main vectors transmitting dengue virus [5]. During 2004–2019, as a result of warmer daily temperatures, their vectorial capacity for the transmission of dengue increased by 25.4% in China [8]. Moreover, it was estimated that both species would expand their distribution over mainland China under future climate scenarios [9]. Another study predicted that potential dengue risk areas in China would further spread to dengue-free regions in the remaining twenty-first century, resulting in more population exposure to dengue risk [10]. Guangdong is a dengue high-risk area, and its incidence rate ranks first among all Chinese provinces [11]. As the capital of Guangdong, Guangzhou has high population density and mobility, which is suitable for the transmission of dengue [12], such that > 70% of national dengue cases in 2014 occurred in this city [13]. Hence, it is necessary to explore the factors affecting the dengue incidence in this high-risk area.

Recently, some epidemiological studies have examined the relationship between extreme weather events and dengue incidence. For instance, a study found that drought conditions may increase dengue risk in Barbados for up to 5 months [14]. Another study conducted in Guangzhou showed that heatwaves and extremely high rainfall may increase the risk of dengue outbreaks by 115–251% after 6 weeks and 173–258% after 6–13 weeks of exposure, respectively [15]. However, the evidence on the association between tropical cyclones and dengue is still insufficient. A previous study preliminarily indicated that tropical cyclones may increase the incidence of dengue in the Pearl River Delta, China [16]. However, this study regarded the Pearl River Delta as a whole region, ignoring the heterogeneity among internal cities. In addition, this work did not explore the exposure-response relationship in a long lag period or quantify the attributable risk of tropical cyclones on the incidence of dengue. Therefore, our study aimed to evaluate the effects of different levels of tropical cyclones on dengue incidence, quantify the dengue risk attributed to tropical cyclones, and identify the vulnerable populations by using time-series data of dengue cases, tropical cyclones, and meteorological variables. Our findings will contribute to a better understanding of the impact of tropical cyclones on dengue incidence and provide much needed evidence for the development of dengue prevention strategies during tropical cyclones.

## Methods

### Study area and period

**Fig. 1:**
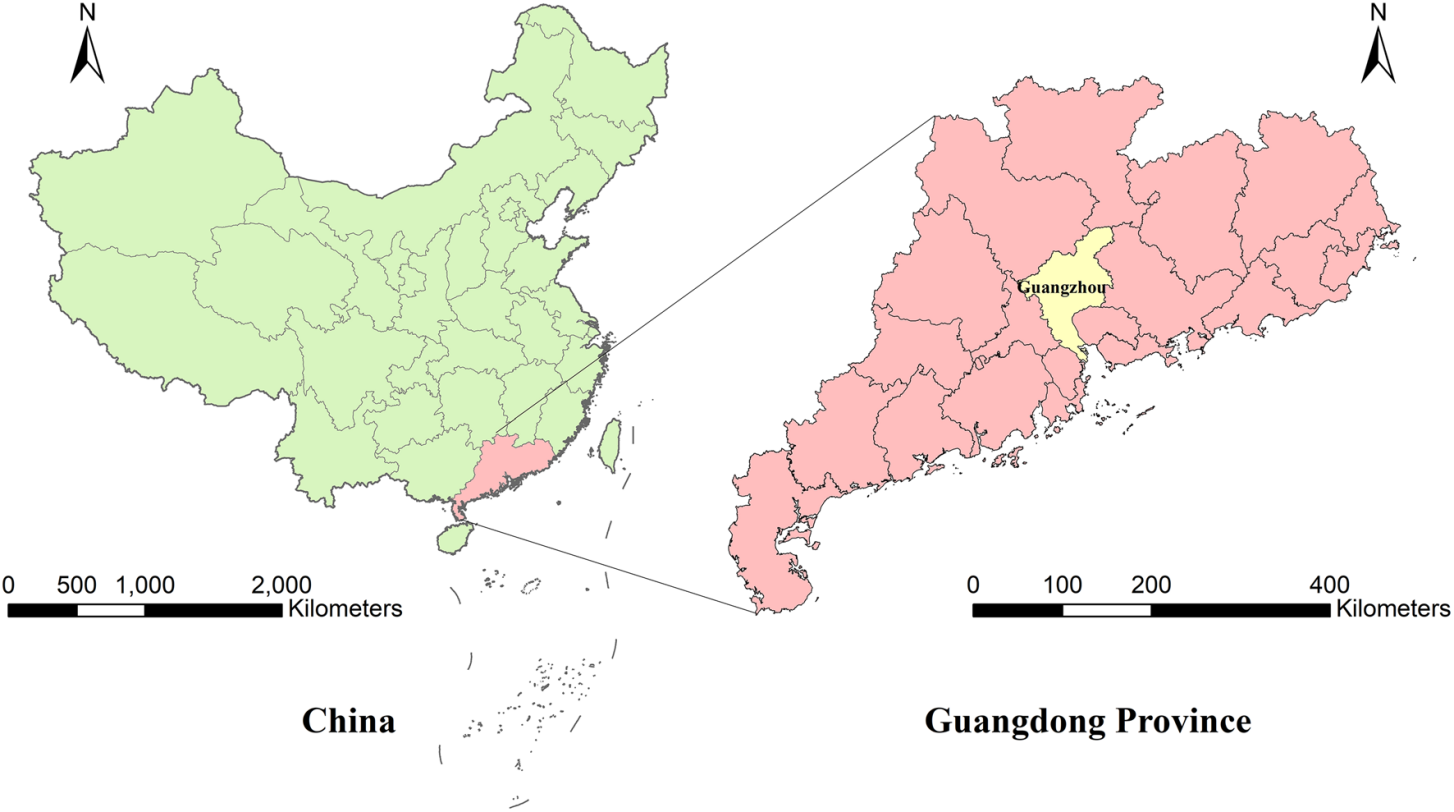
Location of the study area in Guangdong Province, China

Guangzhou (22°39’–23°56’N, 112°57’–114°03’E) is the capital city of Guangdong Province, located in the middle of Guangdong (Fig. 1). Guangzhou has one of the highest population densities in China, with > 15 million residents by 2019 [17]. Meanwhile, Guangzhou is the most important transportation and commercial hub in China. Guangzhou is crossed by the Tropic of Cancer and has a subtropical monsoon climate, which is characterized by hot and rainy summers as well as warm and dry winters. The annual average temperature was 23.1 ℃, the total precipitation 2336 mm, and the average humidity 78.4% in 2019 [17]. Such social and environmental contexts provide favorable conditions for the spread of dengue. In 2014, an unforeseen dengue epidemic occurred in Guangzhou, with > 35,000 people infected [13]. Meanwhile, due to the unique geographical location, Guangzhou is an area frequently affected by tropical cyclones. In this study, we chose June to October as the study period when all tropical cyclones and > 95% of dengue cases (7916/8269) occur in a year.

### Data collection

Dengue is classified as a notifiable B-category infectious disease in China. Under the Law of the People’s Republic of China on the Prevention and Treatment of Infectious Diseases, all cases of dengue should be reported to the local CDC within 24 h after diagnosis [18]. In this study, all dengue cases in Guangzhou reported from June to October 2015–2019 were obtained from the National Notifiable Disease Surveillance System (NDSS) in the Chinese Center for Disease Control and Prevention. All dengue cases were diagnosed by professional medical institutions according to the standard diagnostic criteria for dengue issued by the National Health Commission of China [19, 20]. The individual case information included age, sex, site of onset, and dates (date of onset, diagnosis, and reporting). The weekly number of reported cases was counted in accordance with their date of onset.

Tropical cyclone data were obtained from the China Weather Network (http://typhoon.weather.com.cn/), a meteorological service portal hosted by the China Meteorological Administration. Tropical cyclone information including the generation and disappearance date, path, landing location, and wind speed were obtained. Then, Beaufort wind scale was used to determine the exposure period of tropical cyclones. Specifically, we defined the exposure period as the period from the day when the level 7 wind circle (the average wind speed within the circle was above 13.9–17.1 m/s) of the tropical cyclone landed in Guangzhou to the day when the level 7 wind circle left Guangzhou or the tropical cyclone naturally disappeared in Guangzhou [16]. According to the China Meteorological Administration, tropical cyclones were divided into six intensity levels according to the maximum surface wind speed near their centre, i.e. tropical depression (10.8–17.1 m/s), tropical storm (17.2–24.4 m/s), severe tropical storm (24.5–32.6 m/s), typhoon (32.7–41.4 m/s), severe typhoon (41.5–50.9 m/s), and super typhoon (≥ 51 m/s) [21]. We took the intensity during the exposure period as the tropical cyclone level affecting Guangzhou. Considering no tropical depression affected Guangzhou during the study period, tropical cyclones were reclassified into two levels, namely tropical storm (including tropical storm and severe tropical storm) and typhoon (including typhoon, severe typhoon, and super typhoon).

Weekly meteorological data during the study period were collected from the China Meteorological Data Service Centre (http://data.cma.cn/), including weekly minimum, average, and maximum temperature, cumulative precipitation, average relative humidity, average wind speed, and sunshine hours. The meteorological data used in this study were from the weather station in Tianhe district, Guangzhou, located at 23°13’N and 113°29’E.

### Statistical analysis

A quasi-Poisson generalized linear model combined with a distributed lag non-linear model (DLNM) was employed to quantify the effects of tropical cyclones on dengue incidence. DLNM can examine nonlinear exposure response dependence and lag effects simultaneously by constructing the cross basis [22], which is widely used for estimating the association between environmental exposures and infectious diseases [23, 24]. In this study, cross-basis function was established for tropical cyclones. Specifically, linear function was used to examine the relationship in the dimension of exposure, and polynomial function with four degrees of freedom (df) was used to investigate the relationship in the dimension of lags [25, 26]. Natural cubic spline was used for time and week of year (woy) to control long-term trend and seasonality. Previous studies have discovered that meteorological variables influenced dengue transmission [27–29]; hence, we included weekly average temperature, cumulative precipitation, and average relative humidity in DLNM to adjust for potential effects of these meteorological variables. Considering the delayed transmission of dengue virus in mosquito and human hosts and the incubation period of dengue, we explored a 4-week lag for tropical cyclones when establishing cross-basis functions in the model. The model was fitted as follows:

$${\text{log}}\left[ {E\left( {Y_{{\text{t}}} } \right)} \right] \, = \, \alpha \, + cb\left( {TC_{{{\text{t}},{4}}} ,\beta } \right) \, + ns\left( {WAT,{ 3}} \right) \, + ns\left( {WCP,{ 3}} \right) \, + ns\left( {WARH,{ 3}} \right) \, + ns\left( {time,{ 3}} \right) \, + ns\left( {woy,{ 4}} \right)$$

Here, Y_t_ was the number of weekly dengue cases in week t; α was the intercept; cb(TC_t,4_, β) was the cross-basis matrix for tropical cyclones with coefficient β; TC was a binary variable, weeks with and without tropical cyclones were presented by 1 and 0, respectively. ns(WAT, 3), ns(WCP, 3), and ns(WARH, 3) were natural cubic splines of weekly average temperature, weekly cumulative precipitation, and weekly average relative humidity to control their confounding effects, with the df set as three based on previous studies [30, 31]; ns(time, 3) and ns(woy, 4) were natural cubic splines of time and week of year to control long-term trend and seasonality, respectively. Relative risks (RRs) and 95% confidence intervals (CIs) for dengue incidence associated with tropical cyclones were calculated. Absolute number and the fraction of dengue cases attributable to tropical cyclones were then calculated, with 95% empirical CIs (eCIs) estimated through 1000 Monte Carlo simulations [32]. In addition, we analysed and compared the lag and cumulative effects of tropical storms and typhoons on dengue incidence among subgroups (male, female, < 18 years, 18–59 years, and ≥ 60 years).

We conducted the following sensitivity analyses to examine the robustness of the results: (1) replacing the weekly average temperature with the weekly maximum temperature and minimum temperature; (2) varying the df (2–5) for weekly average temperature, cumulative precipitation, and average relative humidity; (3) varying the df (2–5) for time to adjust for long-term trend; (4) adding the term of first-order lagged variable of residual error to control the autocorrelation; (5) fitting an alternative DLNM model by using negative binomial regression; (6) extending the lag period to 6 weeks to examine the duration of the delayed effects.

All statistical analyses in this study were conducted by using the R software (version 4.1.1). We used “dlnm” package to develop models [33]. The level of statistical significance was set at 0.05 (two tailed).

## Results

**Fig. 2:**
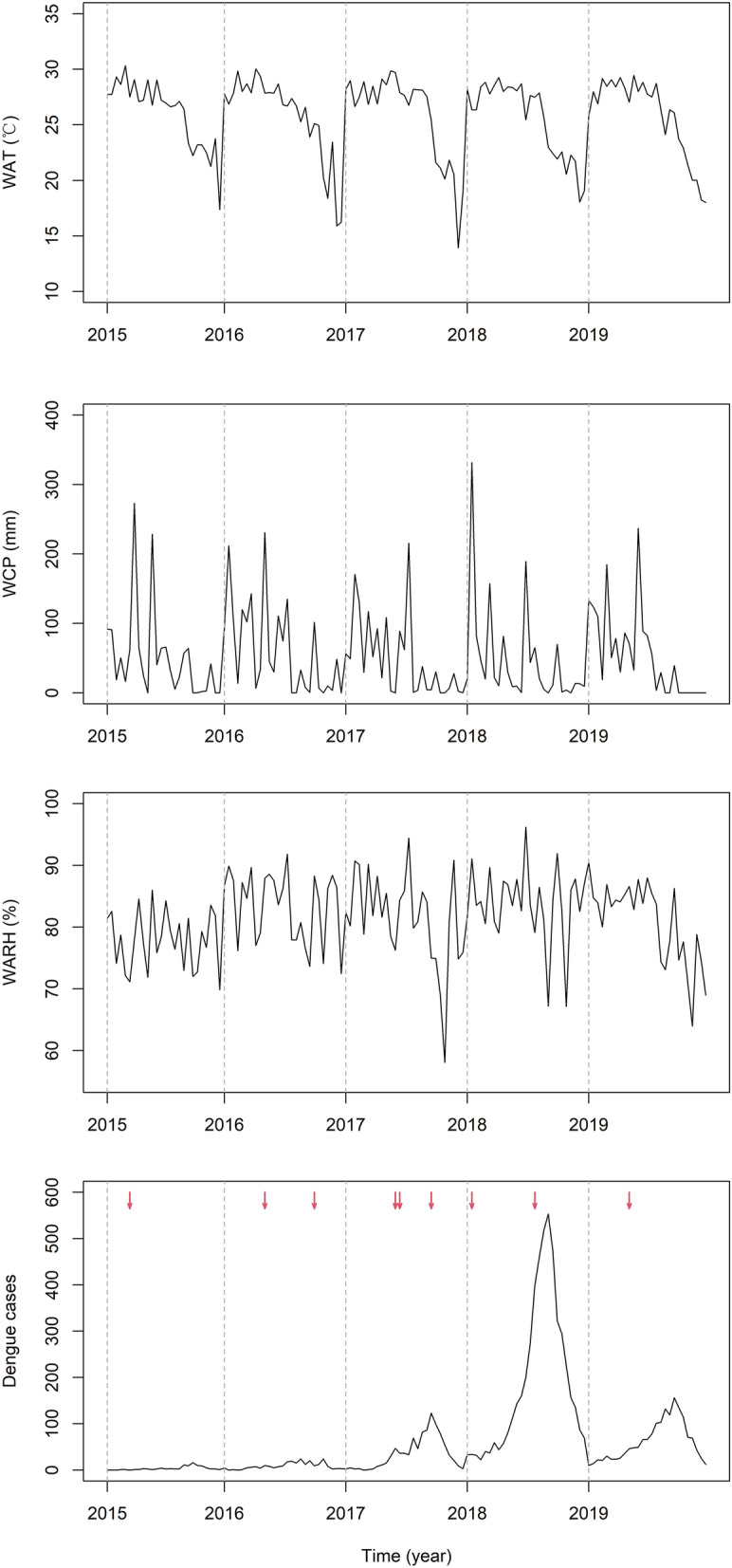
The time-series distribution of weekly average temperature, weekly cumulative precipitation, weekly average relative humidity, and weekly dengue cases during the study period in Guangzhou, China. Tropical cyclone events are marked in the bottom panel with red arrows

Table 1 shows the summary statistics for weekly meteorological conditions and dengue cases in Guangzhou from June to October 2015–2019. There were 7916 dengue cases in Guangzhou over the study period, with a weekly mean of 59 cases. Among them, 54.8% were men and 77.7% aged between 18 and 59 years. The mean weekly average temperature and relative humidity were 25.8 ℃ and 81.5%, respectively. As shown in Fig. 2, there was obvious interannual variation of the dengue cases, which were relatively few in 2015 and 2016 (90 and 236 cases, respectively) and peaked in 2018 (5018 cases). According to the definition of tropical cyclones mentioned above, Guangzhou suffered a total of nine tropical cyclone events during the study period, including three tropical storms and six typhoons. The details of tropical cyclones are shown in Additional file 1: Table S1.

**Table 1:** Descriptive statistics for weekly meteorological factors and dengue cases during study period in Guangzhou, China

	Min	P25	P50	P75	Max
Average temperature (℃)	13.9	23.5	27.1	28.3	30.3
Minimum temperature (℃)	12.0	20.6	24.4	25.1	27.5
Maximum temperature (℃)	17.4	29.0	31.9	33.3	35.9
Cumulative precipitation (mm)	0.0	4.0	30.0	81.0	332.0
Average relative humidity (%)	58.1	77.5	82.5	86.3	96.2
Number of dengue cases					
Male	0.0	2.0	11.5	40.0	301.0
Female	0.0	2.0	8.5	25.8	266.0
< 18 years	0.0	0.0	1.0	4.8	52.0
18–59 years	0.0	3.0	18.0	55.0	411.0
≥ 60 years	0.0	0.0	3.0	9.0	108.0

**Fig. 3:**
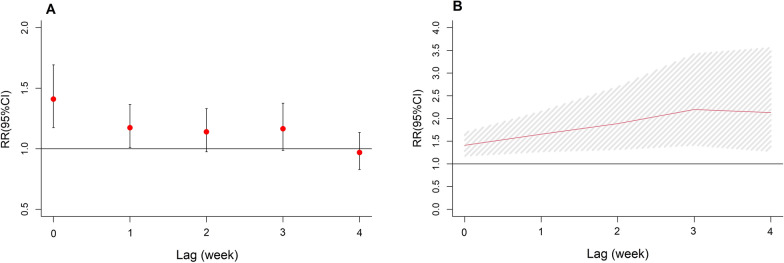
Lag effects (**A**) and cumulative effects (**B**) of tropical cyclones on dengue cases in different lag periods

The lag and cumulative effects of tropical cyclones on dengue incidence are displayed in Fig. 3. Results showed that tropical cyclones were associated with increased risk of dengue incidence in the current week (RR = 1.41, 95% CI: 1.17–1.69) and lag 1 week (RR = 1.17, 95% CI: 1.01–1.37), while no significant association was detected over longer lag weeks (Fig. 3A). The cumulative effects of tropical cyclones on dengue are presented in Fig. 3B. Tropical cyclones were associated with an increased incidence of dengue with a cumulative RR in lag 0–4 weeks equal to 2.13 (95% CI: 1.28–3.56). During the study period, an estimated 500 dengue cases could be explained by tropical cyclones, with the attributable fraction of 6.31% (95% eCI: 1.96–10.16%).

The effects of tropical cyclones on dengue risk with different lags among subgroups are shown in Table 2. Sex-stratified results showed that males were the most vulnerable population with the largest effect estimate occurring in lag 0 week (RR = 1.56, 95% CI: 1.26–1.94), while this association was insignificant among females in all lag weeks. Age-stratified analysis showed that the effects were significant for people aged 18–59 years in lag 0 week (RR = 1.40, 95% CI: 1.13–1.74) and for people > 60 years in lag 0 week (RR = 1.67, 95% CI: 1.14–2.44) and lag 3 week (RR = 1.43, 95% CI: 1.03–2.00). However, tropical cyclones did not significantly change the dengue incidence for people younger than 18 years.

**Table 2:** RRs of tropical cyclones on dengue incidence among different subgroups during the study period in Guangzhou, China

	lag0	lag1	lag2	lag3	lag4	lag0–4
Sex						
Male	1.56(1.26,1.94)^*^	1.20(1.00,1.44)	1.26(1.05,1.52)^*^	1.28(1.05,1.55)^*^	1.04(0.86,1.25)	3.14(1.71,5.77)^*^
Female	1.24(0.98,1.57)	1.15(0.95,1.38)	1.01(0.83,1.23)	1.05(0.84,1.29)	0.90(0.73,1.09)	1.34(0.70,2.58)
Age						
< 18 years	1.34(0.80,2.24)	1.28(0.81,2.02)	1.16(0.73,1.84)	1.34(0.82,2.19)	0.85(0.52,1.37)	2.25(0.54,9.41)
18–59 years	1.40(1.13,1.74)^*^	1.05(0.87,1.27)	1.13(0.94,1.36)	1.15(0.95,1.39)	0.90(0.74,1.10)	1.73(0.97,3.07)
≥ 60 years	1.67(1.14,2.44)^*^	1.42(1.00,2.01)	1.04(0.74,1.46)	1.43(1.03,2.00)^*^	0.94(0.65,1.36)	3.33(1.16,9.55)^*^

^*^Statistically significant

**Fig. 4:**
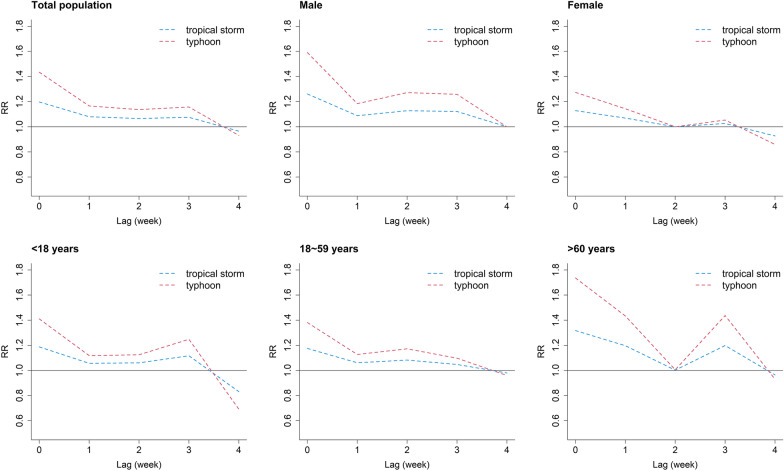
The comparison of lag effects of tropical storms and typhoons on dengue incidence

Figure 4 shows the effects of tropical storms and typhoons on dengue incidence in different lag weeks. Generally, the effects of typhoons were greater than those of tropical storms among the total population and subgroups. The maximum effects of tropical storms and typhoons on dengue incidence both occurred in lag 0 week, with RR = 1.20 (95% CI: 1.09–1.32) and 1.43 (95% CI: 1.18–1.74), respectively. The effects of different levels of tropical cyclones on dengue incidence were more obvious in males and the elderly. The lag and cumulative effects of tropical storms and typhoons on dengue incidence among subgroups are shown in Additional file 1: Table S2.

The effect estimates of tropical cyclones on dengue incidence changed little when replacing the weekly average temperature with the weekly maximum and minimum temperature (Additional file 1: Fig. S1). The effect estimates were similar when changing the df for weekly average temperature, cumulative precipitation, average relative humidity, and time (Additional file 1: Fig. S2). The results changed little when adding the term of first-order lagged variable of residual error (Additional file 1: Fig. S3). Additionally, the results were robust when fitting an alternative DLNM model by using negative binomial regression (Additional file 1: Fig. S4). When extending the lag period to 6 weeks, the results showed that the lag effects of 5–6 weeks were not significant, indicating that a lag of 4 weeks was enough to grasp the short-term effects of tropical cyclone on dengue incidence (Additional file 1: Fig. S5).

## Discussion

Since climate-related disasters continue to adversely affect public health and social development worldwide over recent decades, the research focusing on the impact of extreme weather events on human health is urgently needed [34]. From 2005 to 2018, tropical cyclones affected 478 million people in China and caused direct economic losses of about $ 122 billion. Tropical cyclones have been shown to be associated with a variety of diseases, including infectious and non-communicable diseases [35–37]; however, there is insufficient evidence for the relationship between tropical cyclones and dengue. In this study, we quantified the relative risk of tropical cyclones on dengue in Guangzhou, China, during 2015–2019 using distributed lag nonlinear models. Our results showed that tropical cyclones were positively associated with the dengue incidence among the whole population in a 4-week lag period. Men and the elderly are more vulnerable to the short-term effect of tropical cyclones on dengue incidence than others.

A few studies have also reported an increased risk of dengue after tropical cyclone landfall. For instance, a study in southeast China indicated that tropical cyclones were likely to increase the risk of dengue [37]. Another study found that the landing of hurricanes may trigger an outbreak of dengue in Havana, Cuba, resulting in a serious and long-lasting epidemic [38]. The adverse effect of tropical cyclones on dengue may be realized by affecting the etiological chain of “tropical cyclone-*Aedes*-dengue” [16, 37]. The impact of tropical cyclones on mosquito is multi-pronged. First, the huge energy carried by tropical cyclones may directly cause severe damage to anti-mosquito buildings and facilities (e.g. doors, windows, houses, electricity), thus increasing the possibility of contact between mosquitoes and humans [39]. For instance, tropical cyclones caused > 7000 houses to collapse and 350,000 people to urgently relocate in Guangdong Province in 2014 [40], which may have contributed to the anomalous dengue epidemic. Another study conducted in southwest Florida showed that the lengthy loss of electrical power after Hurricane Irma forced most residents outdoors, increasing their exposure to mosquitoes [41]. In addition, the heavy rainfall carried by tropical cyclones might provide a conducive environment for mosquito breeding. A study suggested that heavy precipitation accompanied by a hurricane led to more breeding reservoirs for the larvae to proliferate, giving rise to the ensuing dengue outbreak [38]. However, our study failed to integrate vector density and biological parameters (e.g. lifespan, extrinsic incubation period, positive rate of dengue virus), thus hindering the inference of the association mechanism between tropical cyclones and dengue incidence. A study in Puerto Rico found that the abundance of *Aedes aegypti* did not increase significantly in vector-controlled communities after the hurricane, and dengue virus was not detected in *Aedes aegypti*, while dengue virus was detected in communities without vector control [42]. It explicitly supports vector control as the primary public health intervention for reducing dengue transmission at the community level [43]. Emergency mosquito control measures including applying insecticides as space spraying during and after tropical cyclones exposure should be used by health authorities [5].

We identified a 1-week lag effect of tropical cyclones on dengue incidence. Similar findings have been reported in many studies on the association between meteorological factors and vector-borne infectious diseases [31, 35]. A study in Guangdong Province, China, demonstrated that tropical storms increased the risk of hand, foot, and mouth disease among children younger than 6 years old after 4 lag days (OR = 1.55, 95% CI: 1.28–1.88) [35]. The reasons for the lag might be complex because of the incubation period of dengue virus, including extrinsic and intrinsic incubation period, as well as the development cycle of mosquitoes after tropical cyclones [44]. Most notably, our previous preliminary research reached a similar conclusion that tropical cyclones have a lag effect on days 5–9 [16]; the current study further claimed that the cumulative effect of tropical cyclones on the dengue incidence could be extended to 4 weeks. This new insight suggests that multifaceted vector control programmes in light of local conditions should be strengthened for at least 1 month after tropical cyclone exposure to achieve maximum effectiveness.

We found that the subgroups vulnerable to the impact of tropical cyclones on dengue were males and the elderly > 60 years old. The specific reasons for this disproportionate impact between sex and age are still unclear. However, one possible explanation for this sex difference in effect estimates is that men are more likely to undertake relief and reconstruction activities after natural disasters (e.g. tropical cyclones, floods). Substandard living conditions and weak awareness of mosquito-borne disease prevention after tropical cyclone exposure may increase the possibility of subjects’ exposure to mosquito bites [39]. In 2020, the elderly aged > 60 years accounted for 18.27% of the total registered residence in Guangzhou [45]. With the surge of the aging population in recent years, there is a call for strengthening the research on the disaster-induced risk assessment for the elderly. Although it has been well proved that the elderly are particularly vulnerable to health risks during extreme weather events [46, 47], few studies examine the dengue risk among the elderly associated with tropical cyclones. This study demonstrated the increased risk of dengue in the elderly after exposure to tropical cyclones. As many have functional limitations and decreased immunity, the elderly with weak post-disaster resilience might be the least able to cope without assistance to prepare and respond. In addition, the shrinking social network of the elderly can also retard the recovery and reconstruction after tropical cyclones [48]. Therefore, the vulnerable subgroups identified in this study should be given more protection against the dengue risk induced by tropical cyclones.

This study also found that the risk of dengue caused by typhoons is stronger than that by tropical storms among all subpopulations. Unfortunately, compared with the 1980–1999 period, a statistically significant increase has been detected in the occurrence of severe and super typhoons from 2000 to 2019 in China [4]. Therefore, if this trend remains unchanged over subsequent decades, it is speculated that the attributable risk of dengue by tropical cyclones will further increase and bring a higher healthcare burden. In addition, it is likely that the latitude where tropical cyclones in the western North Pacific reach their peak intensity has shifted northward [3]. This suggests that we should pay more attention to the northern coastal areas (e.g. northern subtropical cities) that may suffer more major tropical cyclones in the future causing dengue outbreaks to occur more frequently once cases are imported.

Our study has some strengths. First, by applying the DLNM model, we were able to qualify the delayed and cumulative effects of tropical cyclones on dengue. Second, June to October instead of the full year was selected as the study period in our work, which would control confounding effects [26]. Third, this study estimated the attributable risk of dengue caused by tropical cyclones, which, to our knowledge, has not been quantified before. Finally, a variety of sensitivity analyses were performed, which fully proved the robustness of coefficient estimates.

There are several limitations that need to be discussed. First, an ecological design was used, which provided evidence of the association between tropical cyclones and dengue incidence while hindering the causal inference. The exposure levels of tropical cyclones and meteorological factors in this study were obtained at city- rather than individual-level exposure, thereby inevitably leading to ecological fallacy. Second, it should be acknowledged that the results of the single site study may be lack of generalizability. However, given the unique geographical location and high dengue incidence rate of Guangzhou, the current work could also contribute to the limited knowledge of the complex associations between tropical cyclones and dengue. Third, the dengue cases included in this study may be a subset of real cases due to the filtering of asymptomatic cases and suspected cases. However, this bias is likely to be nondifferential and randomly distributed over time [49], so it is considered not to cause large fluctuations in the current results. Fourth, we defined the exposure of tropical cyclones according to the Beaufort wind scale in this study; this method fails to further explore the impact of the tropical cyclone path and the distance from the city centre on the effect estimates. Certainly, this deserves further study once longer time series and more tropical cyclones are included. Finally, as a strict mosquito-borne infectious disease, it is the spread of *Aedes* mosquitoes that has driven expansion of dengue [50]. Dengue is closely related to the survival and development of mosquitoes. The exposure of tropical cyclones may affect the dengue incidence by altering the habitat of mosquitoes. However, we did not consider this effect because high-quality data regarding mosquito density were not available. Meanwhile, we also ignored some socioeconomic confounding factors, such as population immunity level, healthcare services, and population structure changes. In the future, it will be meaningful to extend this study into an integrated “tropical cyclone-mosquito-dengue” system including vector biological parameters and relevant demographic, social, and healthcare variables.

## Conclusion

This study indicates that tropical cyclones are significantly associated with dengue incidence within 4 weeks in Guangzhou, China, with the attributable fraction being 6.31%. Typhoons have a stronger impact on the dengue incidence than tropical storms. Men and people aged > 60 years old appeared to be more vulnerable than the others. Our findings suggest that the strategy to prevent dengue should be combined with the distribution pattern of tropical cyclones. Preventive measures should be strengthened at least 1 month after tropical cyclone exposure, especially for vulnerable groups to reduce the dengue cases associated with tropical cyclones.

## Supplementary Information

**Additional file 1: Table S1**. The detail information of tropical cyclones during the study period. **Table S2**. The lag and cumulative effects of tropical storm and typhoon on dengue incidence among subgroups. **Figure. S1**. The lag effects of tropical cyclones on dengue incidence when changing the meteorological factors in the model. **Figure. S2**. The lag effects of tropical cyclones on dengue incidence when changing the df (2–5) for WAT, WCP, WARH, and time. **Figure. S3**. The lag effects of tropical cyclones on dengue incidence when adding the term of first-order lagged variable of residual error in the model. **Figure. S4**. The lag effects of tropical cyclones on dengue incidence when using negative binomial regression rather than quasi-Poisson regression. **Figure. S5**. Lag effects (A) and cumulative effects (B) of tropical cyclones on dengue incidence within lag 6 weeks.

## Data Availability

The data that support the findings of this study are available from the Chinese Center for Disease Control and Prevention but restrictions apply to the availability of these data, which were used under license for the current study and so are not publicly available. Data are however available from the authors upon reasonable request and with permission of Chinese Center for Disease Control and Prevention.

## Abbreviations

NDSS: National Notifiable Disease Surveillance System
DLNM: Distributed lag non-linear model
df: Degrees of freedom
woy: Week of year
WAT: Weekly average temperature
WCP: Weekly cumulative precipitation
WARH: Weekly average relative humidity
RR: Relative risk
CI: Confidence interval

## References

[1] Guzman O, Jiang H (2021). Global increase in tropical cyclone rain rate. Nat Commun.

[2] Yang W, Hsieh T-L, Vecchi GA (2021). Hurricane annual cycle controlled by both seeds and genesis probability. Proc Natl Acad Sci U S A.

[3] IPCC AR6 WGI. Climate change 2021 the physical science basis. 2021. https://www.ipcc.ch/report/ar6/wg1/downloads/report/IPCC_AR6_WGI_Full_Report.pdf. Accessed 26 Dec 2021.

[4] Cai W, Zhang C, Suen HP, Ai S, Bai Y, Bao J (2021). The 2020 China report of the Lancet Countdown on health and climate change. Lancet Public Health.

[5] World Health Organization. Dengue and severe dengue. 2021. https://www.who.int/news-room/fact-sheets/detail/dengue-and-severe-dengue. Accessed 26 Dec 2021.

[6] Kakarla SG, Bhimala KR, Kadiri MR, Kumaraswamy S, Mutheneni SR (2020). Dengue situation in India: Suitability and transmission potential model for present and projected climate change scenarios. Sci Total Environ.

[7] World Health Organization. Dengue data application. 2021. https://ntdhq.shinyapps.io/dengue5. Accessed 26 Dec 2021.

[8] Cai W, Zhang C, Zhang S, Ai S, Bai Y, Bao J (2021). The 2021 China report of the Lancet Countdown on health and climate change: seizing the window of opportunity. Lancet Public Health.

[9] Liu B, Gao X, Ma J, Jiao Z, Xiao J, Hayat MA (2019). Modeling the present and future distribution of arbovirus vectors *Aedes aegypti* and *Aedes albopictus* under climate change scenarios in Mainland China. Sci Total Environ.

[10] Fan J, Liu Q (2019). Potential impacts of climate change on dengue fever distribution using RCP scenarios in China. Adv Clim Chang Res.

[11] Zhang H, Mehmood K, Chang Y-F, Zhao Y, Lin W, Chang Z (2020). Increase in cases of dengue in China, 2004–2016: a retrospective observational study. Travel Med Infect Dis.

[12] Liu K, Yin L, Zhang M, Kang M, Deng A-P, Li Q-L (2021). Facilitating fine-grained intra-urban dengue forecasting by integrating urban environments measured from street-view images. Infect Dis Poverty.

[13] Oidtman RJ, Lai S, Huang Z, Yang J, Siraj AS, Reiner RC (2019). Inter-annual variation in seasonal dengue epidemics driven by multiple interacting factors in Guangzhou, China. Nat Commun.

[14] Lowe R, Gasparrini A, Van Meerbeeck CJ, Lippi CA, Mahon R, Trotman AR (2018). Nonlinear and delayed impacts of climate on dengue risk in Barbados: a modelling study. PLoS Med.

[15] Cheng J, Bambrick H, Frentiu FD, Devine G, Yakob L, Xu Z (2021). Extreme weather events and dengue outbreaks in Guangzhou, China: a time-series quasi-binomial distributed lag non-linear model. Int J Biometeorol.

[16] Li C, Zhao Q, Zhao Z, Liu Q, Ma W (2021). The association between tropical cyclones and dengue fever in the Pearl River Delta, China during 2013–2018: a time-stratified case-crossover study. PLoS Negl Trop Dis.

[17] Guangzhou Statistics Bureau. Guangzhou Statistical Yearbook 2020. 2019.

[18] National health commission of the People’s Republic of China. Measures for the implementation of the law of the People's Republic of China on the prevention and treatment of infectious diseases. 2018. http://www.nhc.gov.cn/fzs/s3576/201808/58d2b24710c14c2f97ae6de5a8059b73.shtml. Accessed 26 Dec 2021.

[19] Ministry of Health of People's Republic of China. Diagnostic criteria for dengue fever (WS 216-2008). 2008. http://www.nhc.gov.cn/wjw/s9491/200802/38819.shtml. Accessed 26 Dec 2021.

[20] National health commission of the People’s Republic of China. Diagnosis for dengue fever (WS 216-2018). 2018. http://www.nhc.gov.cn/wjw/s9491/201803/d524df26df28453eada8371dc3565818.shtml. Accessed 26 Dec 2021.

[21] China Meteorological Administration. Grade of tropical cyclones (GB/T 19201–2006). 2006. http://zwgk.cma.gov.cn/zfxxgk/gknr/flfgbz/bz/202102/t20210210_2719395.html. Accessed 26 Dec 2021.

[22] Gasparrini A, Armstrong B, Kenward MG (2010). Distributed lag non-linear models. Stat Med.

[23] Yi X, Chang Z, Zhao X, Ma Y, Liu F, Xiao X (2020). The temporal characteristics of the lag-response relationship and related key time points between ambient temperature and hand, foot and mouth disease: a multicity study from mainland China. Sci Total Environ.

[24] Yuan J, Wu Y, Jing W, Liu J, Du M, Wang Y (2021). Association between meteorological factors and daily new cases of COVID-19 in 188 countries: a time series analysis. Sci Total Environ.

[25] Gasparrini A (2014). Modeling exposure-lag-response associations with distributed lag non-linear models. Stat Med.

[26] Liu Z, Lao J, Zhang Y, Liu Y, Zhang J, Wang H (2018). Association between floods and typhoid fever in Yongzhou, China: effects and vulnerable groups. Environ Res.

[27] Akter R, Hu W, Gatton M, Bambrick H, Cheng J, Tong S (2021). Climate variability, socio-ecological factors and dengue transmission in tropical Queensland, Australia: a Bayesian spatial analysis. Environ Res.

[28] Chumpu R, Khamsemanan N, Nattee C (2019). The association between dengue incidences and provincial-level weather variables in Thailand from 2001 to 2014. PLoS ONE.

[29] Nova N, Deyle ER, Shocket MS, MacDonald AJ, Childs ML, Rypdal M (2021). Susceptible host availability modulates climate effects on dengue dynamics. Ecol Lett.

[30] Liu Z, Tong MX, Xiang J, Dear K, Wang C, Ma W (2020). Daily temperature and bacillary dysentery: estimated effects, attributable risks, and future disease burden in 316 Chinese cities. Environ Health Perspect.

[31] Yang Q, Yang Z, Ding H, Zhang X, Dong Z, Hu W (2014). The relationship between meteorological factors and mumps incidence in Guangzhou, China, 2005–2012. Hum Vaccin Immunother.

[32] Gasparrini A, Leone M (2014). Attributable risk from distributed lag models. BMC Med Res Methodol.

[33] Gasparrini A (2011). Distributed lag linear and non-linear models in R: the package dlnm. J Stat Softw.

[34] World Meteorological Organization. The atlas of mortality and economic losses from weather, climate and water extremes (1970–2019). 2021.

[35] Jiao K, Hu W, Ren C, Xu Z, Ma W (2019). Impacts of tropical cyclones and accompanying precipitation and wind velocity on childhood hand, foot and mouth disease in Guangdong Province, China. Environ Res.

[36] Weinberger KR, Kulick ER, Boehme AK, Sun S, Dominici F, Wellenius GA (2021). Association between hurricane Sandy and emergency department visits in New York City by age and cause. Am J Epidemiol.

[37] Zheng J, Han W, Jiang B, Ma W, Zhang Y (2017). Infectious diseases and tropical cyclones in Southeast China. Int J Environ Res Public Health.

[38] Hsieh Y-H, de Arazoza H, Lounes R (2013). Temporal trends and regional variability of 2001–2002 multiwave DENV-3 epidemic in Havana City: did hurricane Michelle contribute to its severity?. Trop Med Int Health.

[39] Seger KR, Roth J, Schnall AH, Ellis BR, Ellis EM (2019). Community assessments for mosquito prevention and control experiences, attitudes, and practices—U.S. Virgin Islands, 2017 and 2018. MMWR Morb Mortal Wkly Rep.

[40] Song L. Yearbook of meteorological disasters in China (2015). 2016.

[41] King RA, Heinig R, Linn P, Lucas KJ (2020). The impact of hurricane Irma on our community and the Collier Mosquito Control District's mission. J Am Mosq Control Assoc.

[42] Barrera R, Felix G, Acevedo V, Amador M, Rodriguez D, Rivera L (2019). Impacts of hurricanes Irma and Maria on populations, aquatic habitats, and mosquito infections with dengue, chikungunya, and Zika viruses in Puerto Rico. Am J Trop Med Hyg.

[43] Kouadio IK, Aljunid S, Kamigaki T, Hammad K, Oshitani H (2012). Infectious diseases following natural disasters: prevention and control measures. Expert Rev Anti Infect Ther.

[44] Kakarla SG, Caminade C, Mutheneni SR, Morse AP, Upadhyayula SM, Kadiri MR (2019). Lag effect of climatic variables on dengue burden in India. Epidemiol Infect.

[45] Guangzhou Statistics Bureau. Guangzhou statistical yearbook 2021. 2021.

[46] Yan M, Wilson A, Dominici F, Wang Y, Al-Hamdan M, Crosson W (2021). Tropical cyclone exposures and risks of emergency medicare hospital admission for cardiorespiratory diseases in 175 urban United States counties, 1999–2010. Epidemiology.

[47] Corley SS, Ornstein KA, Rasul R, Lieberman-Cribbin W, Maisel H, Taioli E (2022). Mental health effects of hurricane Sandy on older adults. J Appl Gerontol.

[48] Wang C, Yarnal B (2012). The vulnerability of the elderly to hurricane hazards in Sarasota, Florida. Nat Hazards.

[49] Zhao Q, Li S, Cao W, Liu D-L, Qian Q, Ren H (2018). Modeling the present and future incidence of pediatric hand, foot, and mouth disease associated with ambient temperature in Mainland China. Environ Health Perspect.

[50] Messina JP, Brady OJ, Golding N, Kraemer MUG, Wint GRW, Ray SE (2019). The current and future global distribution and population at risk of dengue. Nat Microbiol.

