# Supplementary material for: Short-term effects of tropical cyclones on the incidence of dengue: a time-series study in Guangzhou, China
Source: Parasit Vectors. 2022 Oct 6;15:358. doi: 10.1186/s13071-022-05486-2 (PMC9535872; doi:10.1186/s13071-022-05486-2)
Supplement: Supplementary file 1 — Additional file 1: Table S1. The detail information of tropical cyclones during the study period. Table S2. The lag and cumulative effects of tropical storm and typhoon on dengue incidence among subgroups. Figure. S1. The lag effects of tropical cyclones on dengue incidence when changing the meteorological factors in the model. Figure. S2. The lag effects of tropical cyclones on dengue incidence when changing the df (2–5) for WAT, WCP, WARH, and time. Figure. S3. The lag effects of tropical cyclones on dengue incidence when adding the term of first-order lagged variable of residual error in the model. Figure. S4. The lag effects of tropical cyclones on dengue incidence when using negative binomial regression rather than quasi-Poisson regression. Figure. S5. Lag effects (A) and cumulative effects (B) of tropical cyclones on dengue incidence within lag 6 weeks. [file 13071_2022_5486_MOESM1_ESM.docx]

Table S1. The detail information of tropical cyclones during the study period.

| Name | Exposure period | Landing site | Grade |
| --- | --- | --- | --- |
| Linfa | 9^th^–10^th^ Jul, 2015 | Shanwei, Guangdong | Tropical Storm |
| Nida | 2^nd^ Aug, 2016 | Shenzhen, Guangdong | Severe Typhoon |
| Haima | 21^st^ Oct, 2016 | Shanwei, Guangdong | Severe Typhoon |
| Hato | 23^rd^ Aug, 2017 | Zhuhai, Guangdong | Severe Typhoon |
| Pakhar | 27^th^ Aug, 2017 | Zhuhai, Guangdong | Typhoon |
| Khanun | 15^th^ Oct, 2017 | Zhanjiang, Guangdong | Severe Typhoon |
| Ewiniar | 8^th^ Jun, 2018 | Yangjiang, Guangdong | Tropical Storm |
| Mangkhut | 16^th^–17^th^ Sep, 2018 | Jiangmen, Guangdong | Severe Typhoon |
| Wipha | 1^st^ Aug, 2019 | Zhanjiang, Guangdong | Tropical Storm |

Table S2. The lag and cumulative effects of tropical storm and typhoon on dengue incidence among subgroups.

|  |  | lag0 | lag1 | lag2 | lag3 | lag4 | lag0–4 |
| --- | --- | --- | --- | --- | --- | --- | --- |
| Total population | Tropical Storm | 1.20 (1.09–1.32)^*^ | 1.08 (1.00–1.17) | 1.07 (0.98–1.16) | 1.08 (0.98–1.18) | 0.96 (0.89–1.05) | 1.43 (1.09–1.88)^*^ |
|  | Typhoon | 1.43 (1.18–1.74)^*^ | 1.17 (0.99–1.37) | 1.14 (0.97–1.34) | 1.16 (0.96–1.39) | 0.93 (0.78–1.11) | 2.05 (1.19–3.53)^*^ |
| Males | Tropical Storm | 1.26 (1.12–1.42)^*^ | 1.09 (0.99–1.20) | 1.13 (1.02–1.24)^*^ | 1.12 (1.00–1.25)^*^ | 1.00 (0.90–1.11) | 1.74 (1.25–2.41)^*^ |
|  | Typhoon | 1.59 (1.26–2.01)^*^ | 1.18 (0.97–1.44) | 1.27 (1.05–1.55)^*^ | 1.26 (1.01–1.57)^*^ | 1.00 (0.81–1.23) | 3.01 (1.56–5.83)^*^ |
| Females | Tropical Storm | 1.13 (1.00–1.28) | 1.07 (0.97–1.18) | 1.00 (0.90–1.11) | 1.03 (0.91–1.15) | 0.93 (0.83–1.03) | 1.15 (0.82–1.61) |
|  | Typhoon | 1.27 (1.00–1.63) | 1.14 (0.94–1.39) | 1.00 (0.81–1.23) | 1.05 (0.83–1.33) | 0.86 (0.69–1.06) | 1.32 (0.67–2.60) |
| <18 years | Tropical Storm | 1.19 (0.93–1.51) | 1.06 (0.88–1.27) | 1.06 (0.87–1.30) | 1.12 (0.88–1.42) | 0.83 (0.67–1.03) | 1.24 (0.64–2.41) |
|  | Typhoon | 1.41 (0.87–2.28) | 1.12 (0.77–1.62) | 1.13 (0.75–1.68) | 1.25 (0.77–2.02) | 0.69 (0.46–1.05) | 1.53 (0.41–5.80) |
| 18–59 years | Tropical Storm | 1.18 (1.06–1.31)^*^ | 1.06 (0.97–1.16) | 1.08 (0.99–1.18) | 1.05 (0.95–1.16) | 0.98 (0.89–1.08) | 1.39 (1.03–1.87)^*^ |
|  | Typhoon | 1.38 (1.12–1.71)^*^ | 1.13 (0.95–1.34) | 1.17 (0.98–1.40) | 1.10 (0.90–1.35) | 0.96 (0.80–1.16) | 1.93 (1.06–3.50)^*^ |
| ≥60 years | Tropical Storm | 1.32 (1.10–1.58)^*^ | 1.20 (1.04–1.38)^*^ | 1.00 (0.86–1.17) | 1.20 (1.01–1.42)^*^ | 0.96 (0.82–1.14) | 1.83 (1.09–3.06)^*^ |
|  | Typhoon | 1.74 (1.21–2.49)^*^ | 1.43 (1.07–1.91)^*^ | 1.01 (0.73–1.38) | 1.44 (1.02–2.02)^*^ | 0.93 (0.67–1.29) | 3.35 (1.20–9.35)^*^ |

^*^Statistically significant.


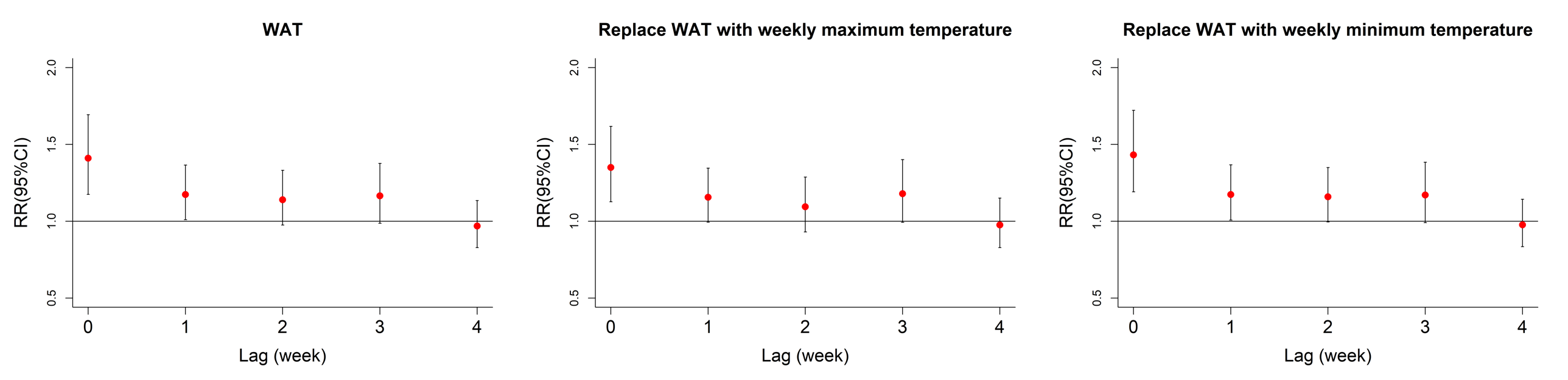


Fig. S1. The lag effects of tropical cyclones on dengue incidence when changing the meteorological factors in the model. WAT: weekly average temperature.





Fig. S2. The lag effects of tropical cyclones on dengue incidence when changing the df (2–5) for WAT, WCP, WARH and time. WAT: weekly average temperature; WCP: weekly cumulative precipitation; WARH: weekly average relative humidity.


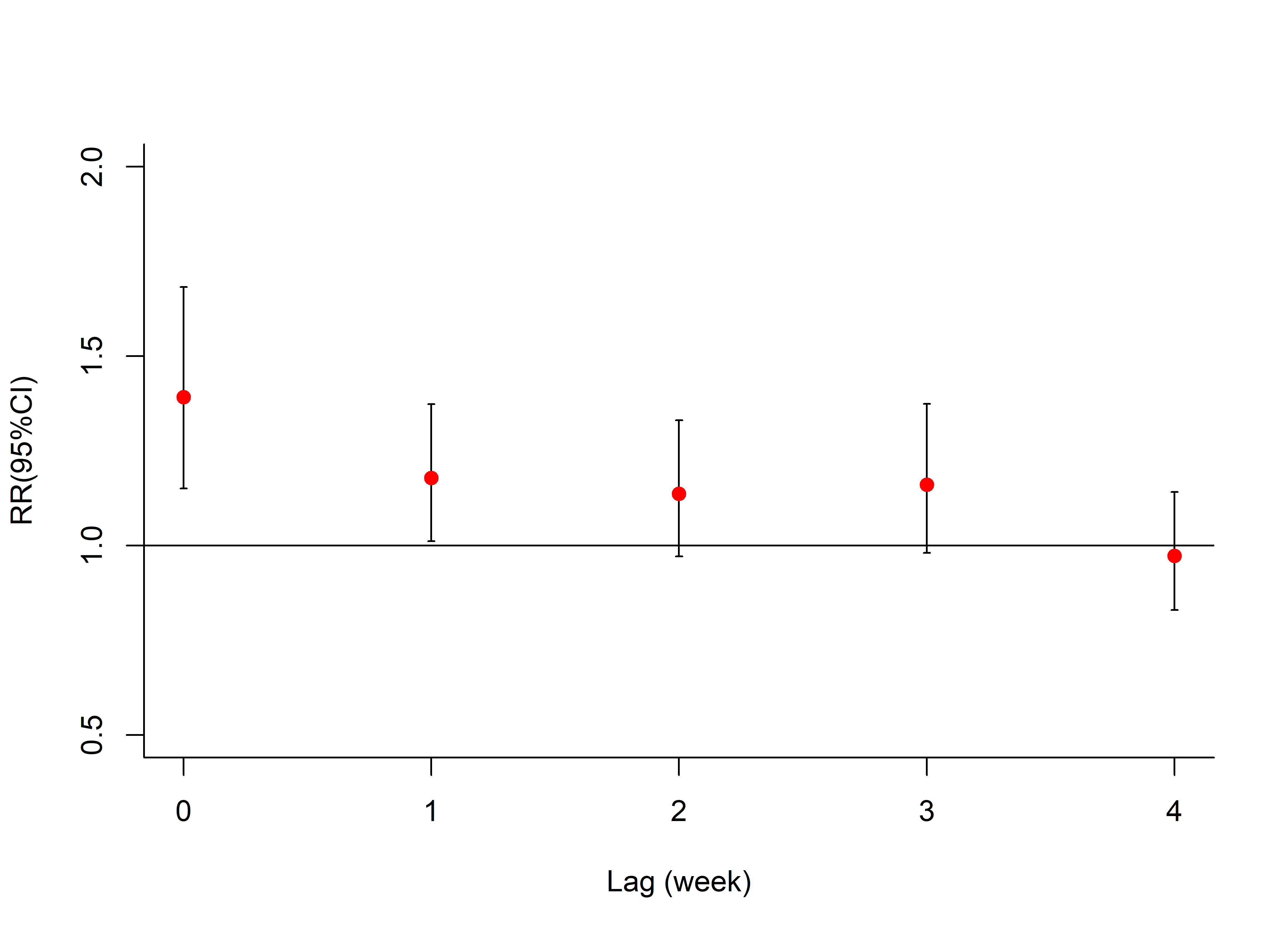


Fig. S3. The lag effects of tropical cyclones on dengue incidence when adding the term of first–order lagged variable of residual error in the model.


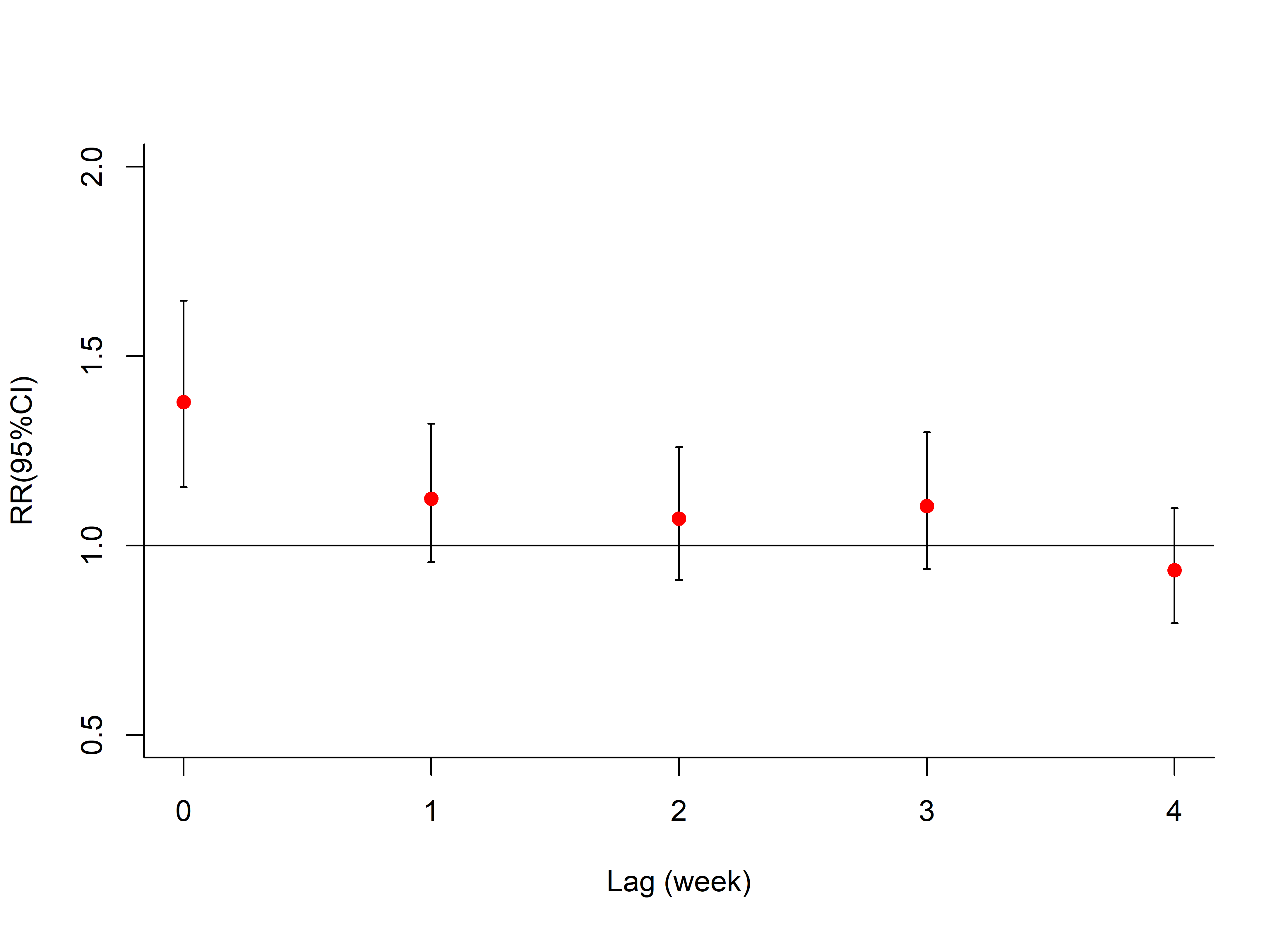


Fig. S4. The lag effects of tropical cyclones on dengue incidence when using negative binomial regression rather than quasi–Poisson regression.


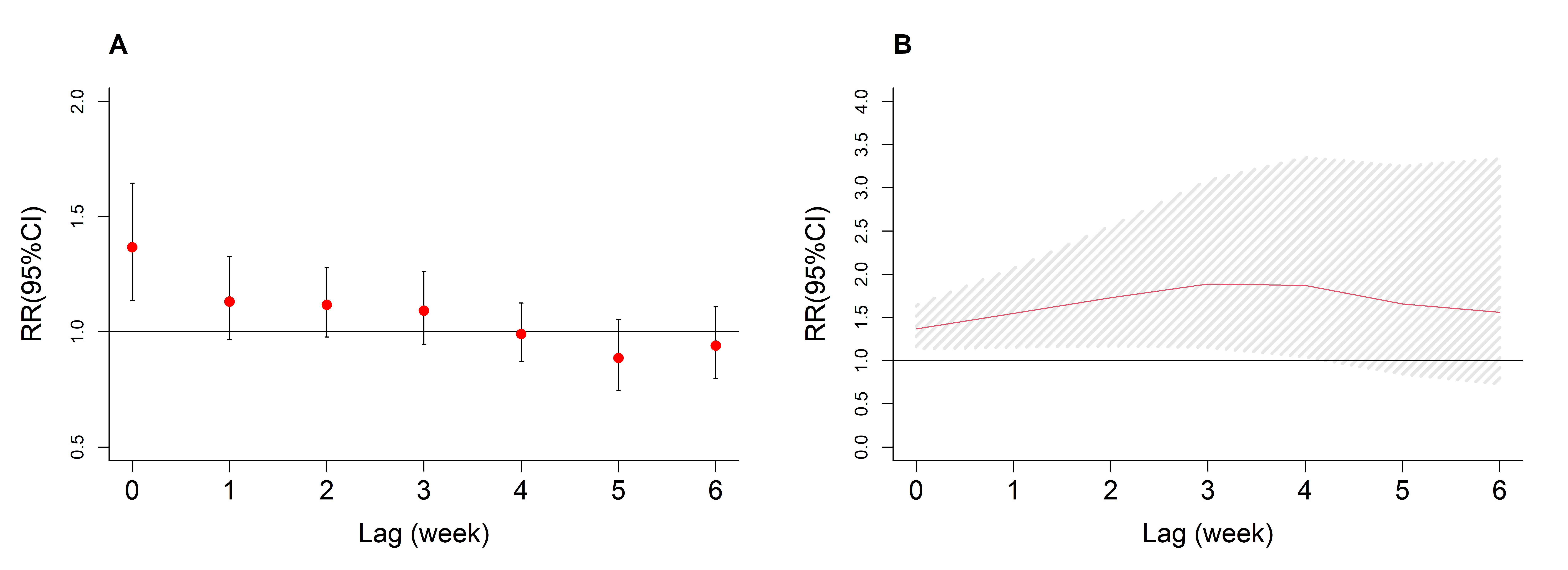


Fig. S5. Lag effects (A) and cumulative effects (B) of tropical cyclones on dengue incidence within lag 6 weeks.
